# Risk of New-onset Stroke in Patients with Type 2 Diabetes with Chronic Kidney Disease on Sodium-glucose Co-transporter-2 Inhibitor Users

**DOI:** 10.1007/s12975-023-01174-0

**Published:** 2023-07-14

**Authors:** Gwo-Ping Jong, Tsung-Kun Lin, Pei-Lun Liao, Jing-Yang Huang, Tsung-Yuan Yang, Lung-Fa Pan

**Affiliations:** 1 Division of Cardiology, Department of Internal Medicine, Chung Shan Medical University Hospital, Taichung, Taiwan, ROC; 2 Institute of Medicine, College of Medicine, Chung Shan Medical University, Taichung, Taiwan, ROC; 3 Department of Pharmacy, Taoyuan Armed Forces General Hospital, Taoyuan, Taiwan, ROC; 4 School of Pharmacy, National Defense Medical Center, Taipei, Taiwan, ROC; 5 Department of Medical Research, Chung Shan Medical University Hospital, Taichung, Taiwan, ROC; 6 Department of Cardiology, Taichung Armed Forces General Hospital, Taichung, Taiwan, ROC; 7 Department of Medical Imaging and Radiological Science, Central Taiwan University of Science and Technology, Takun, Taichung Taiwan, ROC

**Keywords:** New-onset stroke, SGLT2 inhibitor, Type 2 DM, Chronic kidney disease

## Abstract

Clinical studies have investigated the effects of using sodium-glucose co-transporter-2 (SGLT2) inhibitors on the development of new-onset stroke (NOS) in patients with type 2 diabetes (T2D) and chronic kidney disease (CKD), but the findings are inconsistent. This study aimed to examine the association between the use of SGLT2 inhibitors and NOS risk in patients with T2D and CKD. We conducted a nationwide retrospective cohort study using data from the Taiwan Health Insurance Review and Assessment Service database for the years 2004 to 2019. The primary outcome was the risk of incident stroke, which was estimated using hazard ratios (HRs) and 95% confidence intervals (CIs). We used multiple Cox regression modeling to analyze the association between SGLT2 inhibitor use and the risk of stroke in patients with T2D and CKD. In a cohort of 113,710 patients with T2D and CKD who were using SGLT2 inhibitors and 227,420 patients with T2D and CKD who were not using SGLT2 inhibitors, after applying a 1:2 sex- and age-matching strategy, 2,842 and 7,169 NOS events were recorded, respectively. The event rate per 10,000 person-months was 10.60 (95% CI 10.21 to 11.03) for SGLT2 inhibitor users and 13.71 (13.39–14.03) for non-SGLT2 inhibitor users. After adjusting for the index year, sex, age, comorbidities, and concurrent medication, there was a decreased risk of NOS for SGLT2 inhibitor users (adjusted HR 0.80; 95% CI 0.77–0.84) compared with non-SGLT2 inhibitor users. The sensitivity test for the propensity score 1:1-matched analyses showed similar results (adjusted HR 0.80; 95% CI 0.76–0.84). The type of SGLT2 inhibitor subgroup analysis for incident stroke showed consistent results. We concluded that the use of SGLT2 inhibitors in patients with T2D and CKD was associated with significantly low rates of NOS. The significantly low rates of NOS in patients with T2D and CKD were greater among females and less than 50 years patients.

**Supplementary Information:**

The online version contains supplementary material available at 10.1007/s12975-023-01174-0.

## Introduction

The incidence and prevalence of type 2 diabetes (T2D) with chronic kidney disease (CKD) have been on the rise globally over the past two decades, resulting in a significant health burden worldwide [1, 2]. Previous studies have shown that T2D with CKD is associated with an increased risk of cardiovascular diseases, including coronary heart diseases and stroke [3, 4]. Approximately one in six individuals with T2D and CKD experience incident stroke as their initial symptom presentation, and many more go on to have neurological sequelae associated with stroke [5]. Because of this, it is essential to prevent incident stroke in patients with T2D with CKD [6].

Sodium-glucose co-transporter-2 (SGLT2) inhibitors glucose-lowering therapies that target SGLT2 protein and are used in patients with type 2 diabetes [7, 8]. Although these drugs are primarily indicated for diabetes, several studies have examined their use and found that SGLT2 inhibitors may reduce the risk of cardiovascular outcomes in patients with T2DM and CKD, with no evidence of additional safety concerns. Animal studies also have shown a neuroprotective effect of SGLT2 inhibitors, which may play an important role in antioxidant, anti-inflammatory, and antiapoptotic mechanisms [9, 10]. However, the association between SGLT2 inhibitor use and new-onset stroke (NOS) risk in patients with T2D and CKD is inconsistent in clinical studies [11–14]. This study aims to evaluate the risk of NOS associated with the use of SGLT2 inhibitors in patients with T2D and CKD using real-world data from the Taiwanese Bureau of National Health Insurance (BNHI) database.

## Methods

### Study Population and Design

This is a retrospective population-based cohort study that used data from the BNHI database from 2004 to 2019. The database contains anonymized longitudinal medical records that store the claim forms in two tables: a visit table and a prescription table. The visit tables include the patient’s identification numbers, sex, age, three diagnostic codes for outpatient visits and five diagnostic codes for inpatient visits, medications, drug doses, medical expenditures, and hospital, and physician information. The prescription table contains the quantity and expenditure for all the administered drugs, operations, and treatments.

This study was approved by the Ethics Committee of the Chung Shan Medical University Hospital (CS1-21037). Written consent was not obtained from the study participants as only de-identified data were obtained from the Longitudinal Health Insurance Database, and a waiver of patient consent was provided by the Ethics Committee for this study.

### Data Collection

This study included adults (aged > 20 years) with T2D International Classification of Diseases, Tenth Revision, Clinical Modification [ICD-10-CM] code E11) and CKD ICD-10-CM code N18) who were treated with a maximum tolerated labeled dose of an SGLT2 inhibitor and SGLT2 inhibitor non-users. The patients were identified using data from the BNHI database; they were patients admitted to the hospital or outpatients between May 2016 and December 2019.

The study group (SGLT2 inhibitor users) consisted of those patients who received at least one SGLT2 inhibitor prescription continuously for 180 days during the study period. In contrast, the control group (non-SGLT2 inhibitor users) consisted of randomly selected participants with T2D and CKD who did not receive any SGLT2 inhibitor prescriptions throughout the study period.

**Fig. 1:**
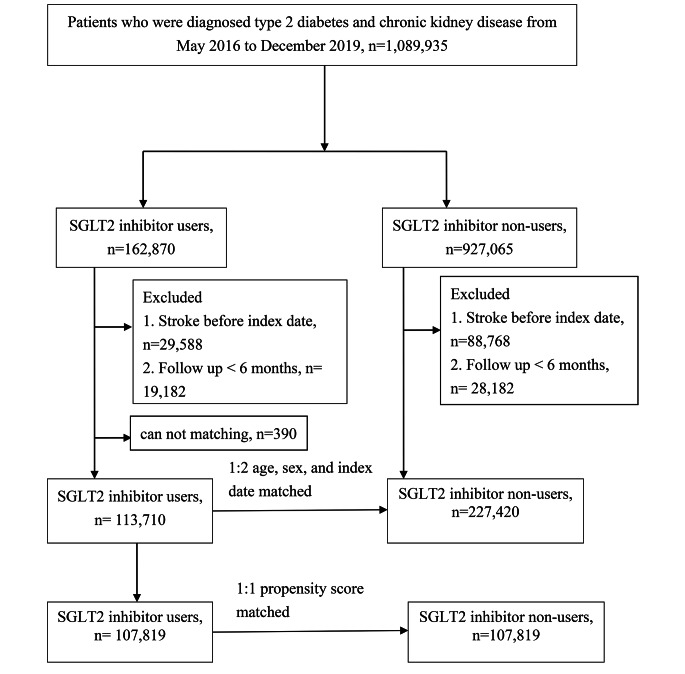
Patient flow chart

The participants (aged > 20 years) had to meet at least one of the following criteria: (1) had two or more outpatient visits within six months with a diagnosis of T2D and CKD, (2) continuously received antidiabetic medication for more than six months during the study period, or (3) had one or more inpatient admissions with a diagnosis of T2D and CKD. Comorbidities related to stroke were recorded according to the ICD-10-CM code and included coronary heart disease (ICD-10-CM code I20–I25), hypertension (ICD-10-CM code I10), hyperlipidemia (ICD-9-CM code E78.1–E78.5), chronic liver disease (ICD-10-CM code K71, K75, K76), chronic obstructive pulmonary disease (ICD-10-CM code J44), atrial fibrillation and flutter (ICD-10-CM code I48), and rheumatoid arthritis (ICD-9-CM code M05). Exclusion criteria included (1) a prior history of stroke before index date and (2) the period of follow up less than 6 months. The index date was defined as the first SGLT-2 inhibitor prescription between May 2016 and December 2019 and the index date for controls was their matched case’s index date. To account for the differences in baseline characteristics and the stroke risk between the SGLT2 inhibitor users and non-SGLT2 inhibitor users (control group), the study group, and control group were matched for age, sex, and T2D duration at a ratio of 1:2. The final study sample comprised 113,710 SGLT2 inhibitor users and 227,420 non-SGLT2 inhibitor users (Fig. 1). A sensitivity analysis using propensity score matching was also performed, with a matching ratio of 1:1 for age, sex, T2D duration, comorbidities, and drug index date (Fig. 1).

### Variables and Outcomes

Patient demographic characteristics were assessed on the index date. The demographic variables included: gender, age, diabetes duration, comorbidities, and concurrent medication. The comorbidity was determined by diagnosis codes 1st time appearing half a year before the index date, and medication use were assessed during a 180-day baseline period. The study endpoint was the development of NOS, defined as the first occurrence of a stroke code (ICD-10-CM codes I60, I61, I62, I63, I65, I66, I67.84, G45, G46) in inpatient or outpatient claim records during follow-up. All patients were followed-up from the index date to the event occurrence date, participant death, or the end of the follow-up period (December 31, 2019), whichever occurred first.

### Statistical Analysis

The number, percentage, and standard deviation of patients meeting each baseline characteristic were reported. Differences in baseline patient characteristics between SGLT2 inhibitor users and non-SGLT2 inhibitor users were examined using t-tests for continuous variables and chi-square tests for categorical variables. We calculated the incidence of study outcomes as the number of patients with NOS after the index date divided by the person-months involved. A log-rank test determined the risk of study outcomes for SGLT2 inhibitor group vs. non-SGLT2 inhibitor group. Univariable and multivariable Cox proportional hazards regression analyses were performed to determine hazard ratios (HRs) with 95% confidence intervals (CIs) of study outcomes for the SGLT2 inhibitor group compared with the non-SGLT2 inhibitor group. The multivariable models were adjusted for important risk factors for developing study events, including age, sex, comorbidities, and concurrent medication. The cumulative risk of study outcomes over time for the SGLT2 inhibitor group compared with the non-SGLT2 inhibitor group was calculated using the Kaplan–Meier method. The survival time was measured as months from index date to NOS or to the end of 2019 if the patient survived, whichever comes first.

A sensitivity analysis was also conducted to test the robustness of our primary findings. Initially, we performed a propensity score matching to balance baseline covariates between SGLT2 inhibitor users and non-SGLT2 inhibitor users. Then, the absolute standardized difference (ASD) was calculated to estimate the difference between the two groups. An ASD < 0.10 implies a negligible difference in the potential confounders between the two groups. Similarly, the impact of each SGLT2 inhibitor user on stroke risk after propensity score matching compared with non-SGLT2 inhibitor user using multivariable logistic regression modeling [15]. The risk of NOS with adjustment the index year, sex, age, comorbidities, and concurrent medication was also assessed. Finally, we also calculated using Kaplan–Meier method for the cumulative risk of study outcomes over time between two groups.

Additionally, we conducted subgroup analyses stratified by sex, age, type of SGLT2 inhibitor, presence of comorbidities, and concurrent medication at baseline for the primary outcomes of NOS. The results are presented as HRs with 95% CIs. Statistical significance was considered at P < 0.05. All statistical calculations were performed using statistical analysis software, version 9.3 (SAS Institute, Inc., Cary, NC, USA).

## Results

### Baseline Characteristics of all Patients

From May 2016 through December 2019, 1,259,539 patients were enrolled in the study. The SGLT2 inhibitor group consisted of 113,710 individuals from the National Health Insurance Research Database, who were newly diagnosed with T2D and CKD. This group was compared with 227,420 control patients who were non-SGLT2 inhibitor users and matched for sex and age at a 1:2 ratio (Fig. 1). Compared with patients in the non-SGLT2 inhibitor group, patients in the SGLT2 inhibitor group had more comorbidities at baseline, except for rheumatoid arthritis (Table 1). There also used more concurrent medication except for proton pump inhibitor (Table 1).

**Table 1:** Baseline characteristics of all patients

	2:1 sex, age matching			After PSM		
	Non- SGLT2	SGLT2	ASD	Non- SGLT2	SGLT2	ASD
N	227,420	113,710		107,819	107,819	
Sex			0.0000			0.0037
Female	99,414 (43.71%)	49,707 (43.71%)		47,020 (43.61%)	47,218 (43.79%)	
Male	128,006 (56.29%)	64,003 (56.29%)		60,799 (56.39%)	60,601 (56.21%)	
Age			0.0000			0.0542
< 50	48,582 (21.36%)	24,291 (21.36%)		23,540 (21.83%)	23,030 (21.36%)	
50–59	62,280 (27.39%)	31,140 (27.39%)		29,681 (27.53%)	29,550 (27.41%)	
60–69	73,498 (32.32%)	36,749 (32.32%)		34,610 (32.10%)	34,858 (32.33%)	
>=70	43,060 (18.93%)	21,530 (18.93%)		19,988 (18.54%)	20,381 (18.90%)	
Comorbidities						
Hypertension	123,747 (54.41%)	66,644 (58.61%)	0.0847	63,109 (58.53%)	62,749 (58.20%)	0.0068
CAD	27,388 (12.04%)	18,224 (16.03%)	0.1149	16,263 (15.08%)	16,506 (15.31%)	0.0063
Hyperlipidemia	126,752 (55.73%)	73,488 (64.63%)	0.1824	69,674 (64.62%)	68,957 (63.96%)	0.0139
Liver disease	24,760 (10.89%)	12,646 (11.12%)	0.0075	11,618 (10.78%)	12,006 (11.14%)	0.0115
Malignancy	13,905 (6.11%)	5539 (4.87%)	0.0546	5051 (4.68%)	5351 (4.96%)	0.0130
COPD	8994 (3.95%)	4536 (3.99%)	0.0018	3977 (3.69%)	4266 (3.96%)	0.0140
Atrial fibrillation and flutter	2662 (1.17%)	1713 (1.51%)	0.0292	1477 (1.37%)	1569 (1.46%)	0.0072
Rheumatoid Arthritis	1810 (0.80%)	707 (0.62%)	0.0208	601 (0.56%)	690 (0.64%)	0.0107
Medication						
NSAIDs	125,976 (55.39%)	65,002 (57.16%)	0.0357	60,782 (56.37%)	61,336 (56.89%)	0.0104
Corticosteroids	42,291 (18.60%)	21,507 (18.91%)	0.0081	19,610 (18.19%)	20,311 (18.84%)	0.0167
PPI	16,549 (7.28%)	7862 (6.91%)	0.0141	7010 (6.50%)	7427 (6.89%)	0.0155
H2 receptor antagonist	59,125 (26.00%)	29,637 (26.06%)	0.0015	27,335 (25.35%)	28,033 (26.00%)	0.0148
Aspirin	48,742 (21.43%)	30,409 (26.74%)	0.1244	27,879 (25.86%)	28,024 (25.99%)	0.0031
Statin	127,254 (55.96%)	79,156 (69.61%)	0.2854	74,605 (69.19%)	73,860 (68.50%)	0.0149
Biguanides	104,614 (46.00%)	68,806 (60.51%)	0.2939	62,122 (57.62%)	63,579 (58.97%)	0.0274
Sulfonylureas	71,427 (31.41%)	47,281 (41.58%)	0.2125	43,888 (40.71%)	43,225 (40.09%)	0.0125
Alpha glucosidase inhibitors	24,399 (10.73%)	21,092 (18.55%)	0.2226	17,827 (16.53%)	18,342 (17.01%)	0.0128
Thiazolidinediones	21,121 (9.29%)	20,865 (18.35%)	0.2649	17,402 (16.14%)	17,640 (16.36%)	0.0060
DPP4	59,662 (26.23%)	45,203 (39.75%)	0.2905	42,268 (39.20%)	40,860 (37.90%)	0.0268
Insullin	44,712 (19.66%)	29,816 (26.22%)	0.1565	26,864 (24.92%)	27,127 (25.16%)	0.0056
GLP-1	3823 (1.68%)	2752 (2.42%)	0.0522	2590 (2.40%)	2493 (2.31%)	0.0059
Type of SGLT-2 i			-			-
Dapagliflozin	0(0%)	58,328(51.30%)		0(0%)	55,272(51.26%)	
Canagliflozin	0(0%)	4654(4.09%)		0(0%)	4466(4.14%)	
Empagliflozin	0(0%)	50,728(44.61%)		0(0%)	48,081(44.595)	

CAD: Coronary artery disease, COPD: Chronic obstructive pulmonary disease, GLP-1:Glucagon-like peptide-1, DPP4: Dipeptidyl peptidase-4. NSAID: Non-steroidal anti-inflammatory drug, PPI: Proton-pump inhibitor. ASD: absolute standardized difference, PSM: propensity score matching

### The Relative Risk of NOS in Patients Matched for Sex and Age at a 1:2 Ratio

**Fig. 2:**
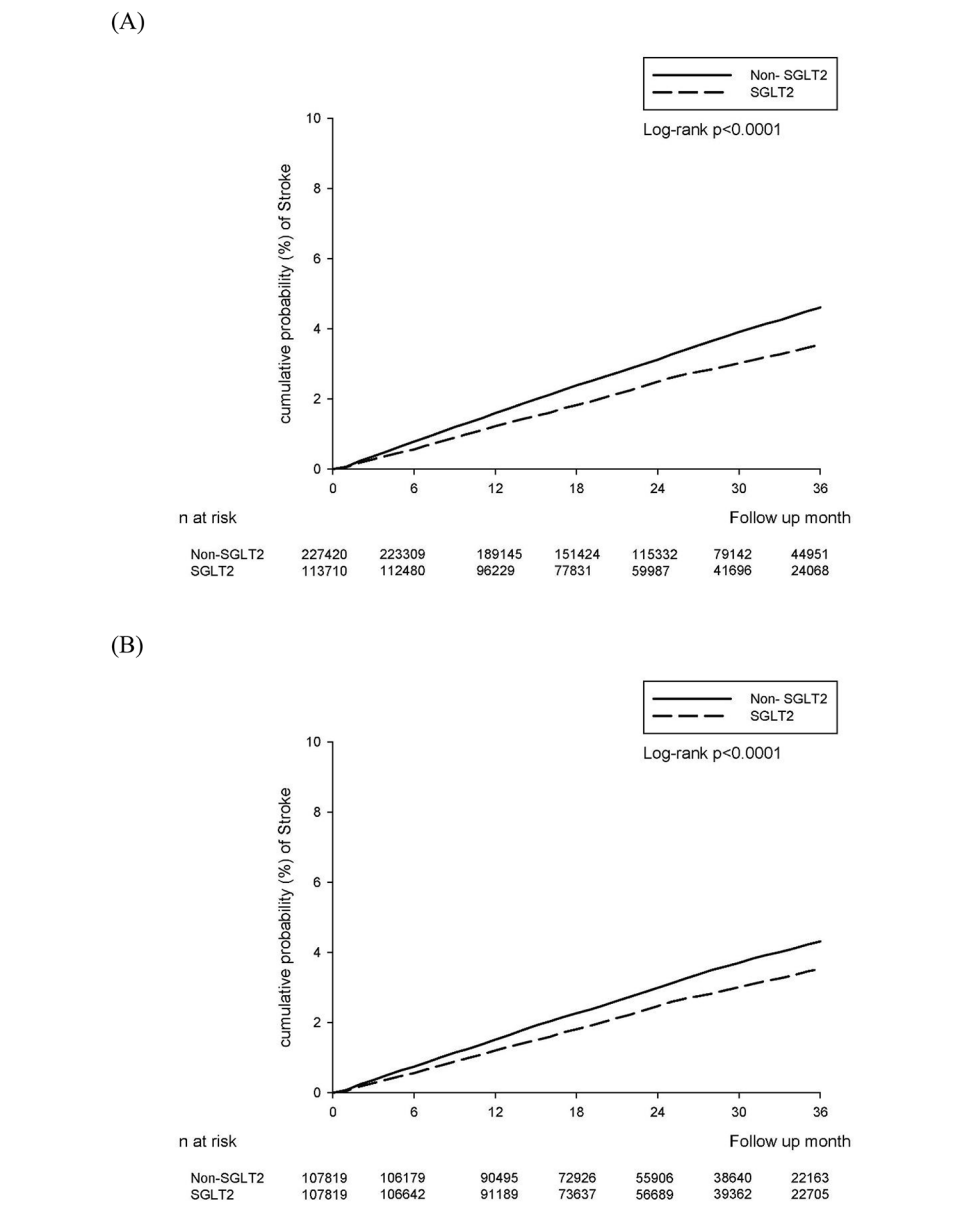
Incident stroke were assessed in time-to-event analyses. (**A**) 2:1 sex and age matching. (**B**) After propensity score matching

The crude incidence rate of NOS was 10.60 per 10 000 person-months (95% CI 10.21–11.03) for SGLT2 inhibitor users compared with 13.71 (95% CI 13.39–14.03) for non-SGLT2 inhibitor users. There was a significantly lower incidence rate of NOS in the SGLT2 inhibitor group compared with the non-SGLT2 inhibitor group (crude HR: 0.77; 95% CI: 0.74–0.81) (Table 2). The results were not substantially changed after adjustments for the index year, sex, age, comorbidities, and concurrent medication at baseline (adjusted HR (aHR): 0.80; 95% CI 0.77–0.84). The effects of SGLT2 inhibitor treatment on NOS were demonstrated in a Kaplan–Meier plot (Fig. 2). Moreover, the cumulative incidence of developing NOS (P < 0.0001) was lower in the SGLT2 inhibitor group than in the non-SGLT2 inhibitor group.

**Table 2:** Association of Sodium-Glucose Cotransporter 2 inhibitors with risk of incident stroke

	2:1 sex age matching		After propensity score matching	
	Non-SGLT2 inhibitors	SGLT2 inhibitors	Non-SGLT2 inhibitors	SGLT2 inhibitors
N	227,420	113,710	107,819	107,819
Follow up person months	5,230,229	2,682,281	2,514,198	2,538,777
New case	7169	2842	3272	2674
Incidence rate*(95% C.I.)	13.71(13.39–14.03)	10.60(10.21–11.03)	13.01(12.58–13.47)	10.53(10.14–10.94)
Crude HR risk (95% C.I.)	reference	0.77(0.74–0.81)	reference	0.81(0.77–0.85)
Adjusted HR* (95% C.I.)†	reference	0.80(0.77–0.84)	reference	0.80(0.76–0.84)

HR: Hazard ratio; SGLT2: Sodium-Glucose Cotransporter 2

*Incidence rate, per 10,000 person-months

† adjusted hazard ratio, the covariates including year of index, sex, age, co-morbidities, and concurrent medication at baseline

### Sensitivity Analysis of the Relative Risk of NOS in a Propensity Score Matching

A sensitivity analysis of the relative risk of NOS in a propensity score matching analysis was conducted. After adjusting the index year, sex, age, comorbidities, and concurrent medication, the results were consistent with the main findings (aHR: 0.80; 95% CI 0.76–0.84; Table 2).

### Subgroup Analyses

Subgroup analyses were conducted to compare the HRs (95% CIs) of study outcomes between the SGLT2 inhibitor group and the non-SGLT2 inhibitor group (Table 3). The results were partly consistent with the main analyses. The type of SGLT2 inhibitor subgroup analysis for incident stroke showed consistent results. Similar findings were also seen for comorbidity with hyperlipidemia (aHR: 0.80; 95% CI 0.77–0.84); chronic liver disease (aHR: 0.87; 95% CI 0.81–0.94); and concurrent medication with a statin (aHR: 0.80; 95% CI 0.77–0.84), biguanide (aHR: 0.75: 95% CI 0.71–0.80), thiazolidinedione (aHR: 0.92; 95% CI 0.86–0.98), and glucagon-like peptide-1 (aHR: 0.79; 95% CI 0.66–0.95). Compared with female and younger patients (aged < 50), male and elderly patients (aged > 50) exhibited a significantly higher risk of stroke. Patients with hypertension, coronary heart disease, and atrial fibrillation were also at significantly higher risks of stroke (aHR: 1.17; 95% CI 1.12–1.22; aHR: 1.14; 95% CI 1.08–1.20; and aHR: 1.62; 95% CI 1.43–1.83, respectively) and concurrent medication with corticosteroids, proton pump inhibitor, aspirin, sulfonylurea, and insulin (aHR 1.10, 95% CI 1.05–1.16; aHR 1.21, 95% CI 1.13–1.30; aHR 1.42, 95% CI 1.36–1.49; aHR 1.06, 95% CI 1.02–1.11; and aHR 1.53, 95% CI 1.47–1.60, respectively). However, an overall null effect of SGLT2 inhibitor on NOS was seen for participants with cancer, chronic obstructive pulmonary disease, and rheumatic arthritis, and concurrent medication with a nonsteroidal anti-inflammatory drug, H2 receptor antagonist, an alpha-glucosidase inhibitor, and dipeptidyl peptidase 4 inhibitor (Table 3).

**Table 3:** Primary outcome from the Cox regression model in the subgroup analysis

	2:1 sex, age matching	aHR(95% CI )		*P* for interaction
		*P* for interaction	1:1 Propensity score matching	
Sex		0.21		0.47
Female	reference		reference	
Male	1.21(1.17–1.27)		1.20(1.14–1.27)	
Age		0.08		0.32
<50	reference		reference	
50–59	1.39(1.3–1.5)		1.38(1.25–1.52)	
60–69	1.86(1.74–1.99)		1.91(1.75–2.09)	
>=70	2.9(2.7–3.12)		3.11(2.83–3.42)	
Type of SGLT2 inhibitor (ref: non-user)				
Dapagliflozin	0.79(0.75–0.84)	0.33	0.79(0.74–0.84)	0.43
Canaglifozin	0.70(0.53–0.92)	0.35	0.7(0.53–0.94)	0.55
Empagliflozin	0.82(0.77–0.87)	0.56	0.82(0.77–0.87)	0.61
Comorbidity(ref: non-comorbidity)				
Hypertension	1.17(1.12–1.22)	0.11	1.18(1.12–1.25)	0.13
CAD	1.14(1.08–1.20)	0.25	1.10(1.03–1.17)	0.22
Hyperlipidemia	0.80(0.77–0.84)	0.18	0.83(0.79–0.88)	0.33
Chronic liver disease	0.87(0.81–0.93)	0.06	0.86(0.79–0.94)	0.25
Malignancy	0.97(0.89–1.05)	0.15	0.96(0.86–1.08)	0.45
COPD	1.00(0.92–1.09)	0.35	0.98(0.88–1.11)	0.95
Atrial fibrillation and flutter	1.62(1.43–1.83)	0.72	1.66(1.43–1.92)	0.68
Rheumatoid Arthritis	0.93(0.75–1.16)	0.45	0.84(0.60–1.17)	0.22
Medication(reference: non-medication)				
NSAIDs	1.03(0.99–1.08)	0.09	1.07(1.01–1.13)	0.20
Corticosteroids	1.10(1.05–1.16)	0.12	1.15(1.08–1.22)	0.28
PPI	1.21(1.13–1.30)	0.38	1.15(1.05–1.27)	0.55
H2 receptor antagonists	1.02(0.97–1.06)	0.18	1.03(0.97–1.09)	0.38
Aspirin	1.42(1.36–1.49)	0.05	1.45(1.37–1.53)	0.45
Statin	0.80(0.77–0.84)	0.09	0.82(0.78–0.87)	0.65
Biguanides	0.75(0.71–0.78)	0.12	0.79(0.75–0.83)	0.55
Sulfonylureas	1.06(1.02–1.11)	0.08	1.10(1.04–1.16)	0.14
Alpha glucosidase inhibitors	0.98(0.92–1.04)	0.19	1.02(0.95–1.08)	0.35
Thiazolidinediones	0.92(0.86–0.98)	0.33	0.92(0.85–0.98)	0.28
DPP4	1.03(0.99–1.08)	0.65	1.03(0.98–1.09)	0.85
Insulin	1.53(1.47–1.60)	0.68	1.52(1.44–1.61)	0.88
GLP-1 inhibitors	0.79(0.66–0.95)	0.09	0.77(0.62–0.95)	0.11

CAD: Coronary artery disease, COPD: Chronic obstructive pulmonary disease, GLP-1:Glucagon-like peptide-1, DPP4: Dipeptidyl peptidase-4. NSAID: Non-steroidal anti-inflammatory drug, PPI: Proton-pump inhibitor

## Discussion

The study presented showed that the risk of experiencing the NOS in patients with T2D and CKD during follow-up period is lower among SGLT2 inhibitor users than in SGLT2 inhibitor non-users. The results of the study also indicated a lower rate of incident stroke in patients with T2D and CKD was greater in female and younger (< 50 years) patients. This study also demonstrated that type of SGLT2 inhibitor subgroup analysis for NOS showed consistent results.

Previous studies on the association between SGLT2 inhibitors and NOS in patients with T2D and CKD have been inconsistent [11–14]. A recent study found a trend toward lower rates of stroke in patients with the lowest estimated glomerular filtration rate (less than 60 mL/min/1.73 m2) (HR: 0.77; 95% CI 0.55–1.08) who used SGLT2 inhibitors [16], but no study has yet shown a lower risk of total stroke in patients with T2D and CKD. However, a meta-analysis did indicate that the effects of SGLT2 inhibitors on total stroke varied depending on baseline estimated glomerular filtration rate, with the greatest protection seen in patients with the lowest estimated glomerular filtration rate (less than 45 mL/min/1.73 m2) [17]. Our study showed that there was evidence that SGLT2 inhibitors affected total stroke in patients with T2D and CKD.

The mechanisms behind the protective effects of SGLT2 inhibitors on cardiovascular disease are not fully understood but may involve reductions in glucose, systolic blood pressure, and arteriosclerosis, as well as protective effects on the heart and kidneys [9, 10, 18–20]. In vitro data suggest that SGLT2 inhibitors improve glucose-induced vascular dysfunction by reducing inflammation and oxidative stress, reversing pro-inflammatory phenotypes, and glucotoxicity in diabetic rats [21].

It is possible that previous studies did not see similar functional effects of SGLT2 inhibitors. However, this current study indicates that canagliflozin, dapagliflozin, and empagliflozin have similar effects in decreasing the number of stroke events. The exact mechanisms behind the beneficial effect of SGLT2 inhibitors on stroke risk are not clear but may involve a reduction in incident atrial fibrillation and atrial flutter. Previous studies and meta-analyses have shown that SGLT2 inhibitors can reduce the risk of incident atrial fibrillation and atrial flutter in patients with T2D and therefore decrease the risk of stroke [17, 22].

Previous epidemiologic studies reveal a clear age-by-sex interaction in stroke incidence [23–25]. A retrospective cohort study from the US health insurance database between 2001 and 2014 including 5.8 million participants found that women had a higher stroke incidence below 44 years of age [25]. Moreover, there are also an epidemiological study show that the trend in decreasing age at diagnosis for stroke and its risk factors appears to be more pronounced among women [26]. The results of this study indicated a decreased risk of incident stroke in patients with T2D and CKD was greater in female and younger (< 50 years) patients. Therefore, earlier identification of stroke risk factors and use SGLT2 inhibitor in younger women with T2D and CKD may provide opportunity to preventive stroke.

The strengths of our study included its population-based nature and large sample size. Our findings were tested using propensity score matching to control for potential confounders, which made our hypothesis feasible. Our study is the first one to provide an association between the use of SGLT2 inhibitor on total stroke risk in patients with T2D and CKD. We found a statistically significant decrease in the risk of NOS among patients who are SGLT2 inhibitor users.

There are several limitations of our investigation that must be noted. First, the study outcome was defined as stroke and comorbidities diagnosis recorded by physicians and were completely dependent on the ICD-10 CM codes; therefore, it is unclear that how our findings can be generalized to patients in different areas of the world. Second, the present study has a retrospective design and the information on several unmeasured confounders, including body mass index, smoking, alcohol intake, and labortory data such as glomerular filtration rate and urinary albumin-to-creatinine ratio, is not available in the National Health Insurance Research Database. However, considering the magnitude and significance of the observed effects, it is unlikely that these limitations compromised the results. Third, the process of stroke in patients who developed NOS in this study would have started many years before the diagnoses, and NOS may have coexisted with the process of T2D and CKD for which SGLT2 inhibitors were used. Thus, the cause-and-effect relationships between NOS and SGLT2 inhibitors cannot be determined in this study. Hence, further a prospective randomized control trial is needed for more detail. Fourth, Diabetes Millitus duration is a strong risk factor for renal function [27]. This is a limitation in this study that T2D duration is not available in the National Health Insurance Research Database. However, because the data we used was population-based data, we assumed that there were no differences among the two groups. Fifth, the National Health Insurance Research Database contained mostly Taiwanese patients. Therefore, the result is difficult to generalize globally.

## Summary

We concluded that the risk of experiencing the NOS during follow-up period is significantly lower among SGLT2 inhibitor users than in SGLT2 inhibitor non-users in patients with T2D and CKD. The lower rate of NOS in patients with T2D and CKD was greater among female and less than 50 years patients. No differences between the type of SGLT2 inhibitor and stroke risk was also observed in this study. Further efforts are necessary to maximize the potential population benefit of these therapies in high-risk populations.

## Electronic Supplementary Material

Below is the link to the electronic supplementary material.

Supplementary Material 1

Supplementary Material 2

## Abbreviations

ASD: absolute standardized difference
BNHI: Bureau of National Health Insurance
CI: confidence interval
CKD: chronic kidney disease
HR: hazard ratio
ICD-10-CM: International Classification of Diseases, Tenth Revision, Clinical Modification
NOS: new-onset stroke
SGLT2: sodium-glucose co-transporter-2
T2D: type 2 diabetes
