# Supplementary material for: Risk of New-onset Stroke in Patients with Type 2 Diabetes with Chronic Kidney Disease on Sodium-glucose Co-transporter-2 Inhibitor Users
Source: Transl Stroke Res. 2023 Jul 14;15(6):1098–107. doi: 10.1007/s12975-023-01174-0 (PMC11522140; doi:10.1007/s12975-023-01174-0)
Supplement: Supplementary file 2 — Supplementary Material 2 [file 12975_2023_1174_MOESM2_ESM.docx]

Supplement

Table 2. The number of the original population with SGLT2 inhibitor users in patients with DM and CKD diagnoses

|  | Number |
| --- | --- |
| Dapagliflozin | 83878(51.50%) |
| Canaglifozin | 6987(4.29%) |
| Empagliflozin | 72005(44.21%) |
